# Tetra­kis[(4-meth­oxy­carbon­yl)anilinium] hexa­chloridostannate(IV) dichloride

**DOI:** 10.1107/S1600536811003692

**Published:** 2011-02-02

**Authors:** Wenzhi Xiao, Ruiting Xue, Yansheng Yin

**Affiliations:** a Department of Physics and Mathematics, Hunan Institute of Engineering, Xiangtan 411104, People’s Republic of China; b Institute of Material Science and Engineering, Ocean University of China, Qingdao, Shandong 266100, People’s Republic of China

## Abstract

The asymmetric unit of the title compound, (C_8_H_10_NO_2_)_4_[SnCl_6_]Cl_2_, contains two (4-meth­oxy­carbon­yl)anilinium cations, one chloride anion and one half of a hexa­chlorido­stannate(IV) dianion situated on a twofold rotation axis. All aminium H atoms are involved in N—H⋯Cl hydrogen bonding, which consolidate the crystal packing along with weak C—H⋯O inter­actions.

## Related literature

For general background to inorganic–organic hybrid compounds, see: Zhang *et al.* (2009 ▶); Descalzo *et al.* (2006 ▶); Li *et al.* (2007 ▶), Sanchez *et al.* (2005 ▶).
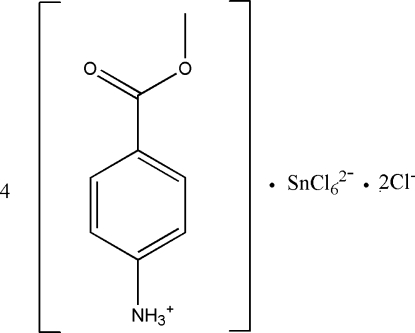


## Experimental

### 

#### Crystal data

(C_8_H_10_NO_2_)_4_[SnCl_6_]Cl_2_
*M* *_r_* = 1010.97
Monoclinic, 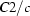

*a* = 30.748 (3) Å
*b* = 7.1172 (8) Å
*c* = 22.113 (2) Å
β = 119.424 (2)°
*V* = 4215.0 (7) Å^3^
*Z* = 4
Mo *K*α radiation
μ = 1.16 mm^−1^
*T* = 298 K
0.50 × 0.46 × 0.46 mm

#### Data collection

Bruker SMART CCD area-detector diffractometer
Absorption correction: multi-scan (*SADABS*; Sheldrick, 1996 ▶) *T*_min_ = 0.594, *T*_max_ = 0.617
10221 measured reflections
3719 independent reflections
2969 reflections with *I* > 2σ(*I*)
*R*_int_ = 0.035

#### Refinement

*R*[*F* ^2^ > 2σ(*F* ^2^)] = 0.031
*wR*(*F* ^2^) = 0.090
*S* = 1.01
3719 reflections
245 parameters
H-atom parameters constrained
Δρ_max_ = 0.51 e Å^−3^
Δρ_min_ = −0.44 e Å^−3^

### 

Data collection: *SMART* (Bruker, 2007 ▶); cell refinement: *SAINT* (Bruker, 2007 ▶); data reduction: *SAINT*; program(s) used to solve structure: *SHELXS97* (Sheldrick, 2008 ▶); program(s) used to refine structure: *SHELXL97* (Sheldrick, 2008 ▶); molecular graphics: *XP* in *SHELXTL* (Sheldrick, 2008 ▶); software used to prepare material for publication: *SHELXL97*.

## Supplementary Material

Crystal structure: contains datablocks global, I. DOI: 10.1107/S1600536811003692/cv5035sup1.cif

Structure factors: contains datablocks I. DOI: 10.1107/S1600536811003692/cv5035Isup2.hkl

Additional supplementary materials:  crystallographic information; 3D view; checkCIF report

## Figures and Tables

**Table 1:** Hydrogen-bond geometry (Å, °)

*D*—H⋯*A*	*D*—H	H⋯*A*	*D*⋯*A*	*D*—H⋯*A*
N1—H1*A*⋯Cl3	0.89	2.78	3.659 (4)	170
N2—H2*B*⋯Cl3	0.89	2.71	3.479 (3)	145
N2—H2*C*⋯Cl4	0.89	2.21	3.098 (4)	177
N1—H1*B*⋯Cl4^i^	0.89	2.29	3.155 (4)	165
N1—H1*C*⋯Cl4^ii^	0.89	2.22	3.092 (4)	166
N2—H2*A*⋯Cl1^iii^	0.89	3.01	3.482 (3)	115
C3—H3⋯O4^iv^	0.93	2.39	3.148 (6)	139
C15—H15⋯O2^v^	0.93	2.38	3.130 (5)	138

Symmetry codes: (i) 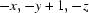; (ii) 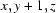; (iii) 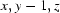; (iv) 
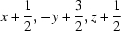
; (v) 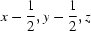.

**Fig. 1.**
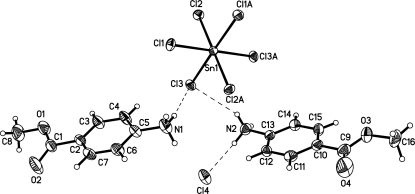
A portion of the title compound, showing 30% probability displacement ellipsoids and the atom-numbering scheme in asymmetric unit [symmetry code (A): -x, y, -z + 1/2]. Dashed lines denote N—H···Cl hydrogen bonds.

## supplementary crystallographic information

### Comment

Considerable attention has been devoted to inorganic-organic hybrid materials over recent years (Zhang *et al.*, 2009). These hybrid materials have potential applications in many areas including gas storage, separation, catalysis, magnetism, optics as well as electrical conductivity (Descalzo *et al.*, 2006; Li *et al.*, 2007; Sanchez *et al.*, 2005]. Herein we report the structure of the title compound (Fig.1.),

This title compound contains SnCl_6_ inorganic anions, organic cations and dissociated chloride anions. The SnCl_6_ inorganic anion adopts a regular octahedron geometry, with average Sn—Cl distance of 2.4262 Å. In the organic cation, the dihedral angle between the ester group and the phenyl ring is 14.86(0.19)°.

In the crystal structure, intermolecular N—H···Cl and C—H···O hydrogen bonds (Table 1) link cations and anions into layers with alternating inorganic and organic species.

### Experimental

4-Aminobenzoic acid (10 mmol) was dissolved to acid methanol solution (10 ml). Ten minutes later, a methanol solution (10 ml) of tin tetrachloride(5 mmol) was added with stirring. The mixture was stirred for 4 h. Crystals of the title compound suitable for X-ray analysis were grown from the saturation ethanol solution after about two weeks.

### Refinement

All H-atoms were positioned geometrically and refined using a riding model, with C—H = 0.96 Å (methyl), 0.93 Å (aromatic), N—H =0.89 Å (ammonium) and *U*_iso_(H) =1.5*U*_eq_(C), *U*_iso_(H) =1.2*U*_eq_(C),*U*_iso_(H) =1.5*U*_eq_(N)

### Crystal data

**Table:**

(C_8_H_10_NO_2_)_4_[SnCl_6_]Cl_2_	*F*(000) = 2040
*M**_r_* = 1010.97	*D*_x_ = 1.593 Mg m^−^^3^
Monoclinic, *C*2/*c*	Mo *K*α radiation, λ = 0.71073 Å
*a* = 30.748 (3) Å	Cell parameters from 4623 reflections
*b* = 7.1172 (8) Å	θ = 2.7–27.7°
*c* = 22.113 (2) Å	µ = 1.16 mm^−^^1^
β = 119.424 (2)°	*T* = 298 K
*V* = 4215.0 (7) Å^3^	Block, yellow
*Z* = 4	0.50 × 0.46 × 0.46 mm

### Data collection

**Table:**

Bruker SMART CCD area-detector diffractometer	3719 independent reflections
Radiation source: fine-focus sealed tube	2969 reflections with *I* > 2σ(*I*)
graphite	*R*_int_ = 0.035
φ and ω scans	θ_max_ = 25.0°, θ_min_ = 1.5°
Absorption correction: multi-scan (*SADABS*; Sheldrick, 1996)	*h* = −29→36
*T*_min_ = 0.594, *T*_max_ = 0.617	*k* = −7→8
10221 measured reflections	*l* = −26→25

### Refinement

**Table:**

Refinement on *F*^2^	Secondary atom site location: difference Fourier map
Least-squares matrix: full	Hydrogen site location: inferred from neighbouring sites
*R*[*F*^2^ > 2σ(*F*^2^)] = 0.031	H-atom parameters constrained
*wR*(*F*^2^) = 0.090	*w* = 1/[σ^2^(*F*_o_^2^) + (0.045*P*)^2^ + 5.2918*P*] where *P* = (*F*_o_^2^ + 2*F*_c_^2^)/3
*S* = 1.01	(Δ/σ)_max_ < 0.001
3719 reflections	Δρ_max_ = 0.51 e Å^−^^3^
245 parameters	Δρ_min_ = −0.44 e Å^−^^3^
0 restraints	Extinction correction: *SHELXL97* (Sheldrick, 2008), Fc^*^=kFc[1+0.001xFc^2^λ^3^/sin(2θ)]^-1/4^
Primary atom site location: structure-invariant direct methods	Extinction coefficient: 0.00204 (13)

### Special details

**Table:**

Geometry. All e.s.d.'s (except the e.s.d. in the dihedral angle between two l.s. planes) are estimated using the full covariance matrix. The cell e.s.d.'s are taken into account individually in the estimation of e.s.d.'s in distances, angles and torsion angles; correlations between e.s.d.'s in cell parameters are only used when they are defined by crystal symmetry. An approximate (isotropic) treatment of cell e.s.d.'s is used for estimating e.s.d.'s involving l.s. planes.
Refinement. Refinement of *F*^2^ against ALL reflections. The weighted *R*-factor *wR* and goodness of fit *S* are based on *F*^2^, conventional *R*-factors *R* are based on *F*, with *F* set to zero for negative *F*^2^. The threshold expression of *F*^2^ > σ(*F*^2^) is used only for calculating *R*-factors(gt) *etc*. and is not relevant to the choice of reflections for refinement. *R*-factors based on *F*^2^ are statistically about twice as large as those based on *F*, and *R*- factors based on ALL data will be even larger.

### Fractional atomic coordinates and isotropic or equivalent isotropic displacement parameters (Å^2^)

**Table:**

	*x*	*y*	*z*	*U*_iso_*/*U*_eq_	
Sn1	0.0000	0.88525 (4)	0.2500	0.03147 (14)	
Cl1	0.05414 (3)	1.12542 (12)	0.24657 (5)	0.0448 (2)	
Cl2	0.04668 (3)	0.88401 (13)	0.37614 (4)	0.0446 (2)	
Cl3	0.05329 (3)	0.64043 (12)	0.24497 (5)	0.0452 (2)	
Cl4	0.01937 (4)	0.27991 (19)	0.05504 (6)	0.0745 (4)	
N1	0.05296 (13)	0.8682 (5)	0.09640 (19)	0.0681 (11)	
H1A	0.0488	0.8145	0.1295	0.102*	
H1B	0.0354	0.8058	0.0568	0.102*	
H1C	0.0425	0.9868	0.0908	0.102*	
O1	0.28945 (11)	0.8266 (5)	0.23899 (19)	0.0801 (9)	
O2	0.27319 (15)	0.8654 (6)	0.1308 (2)	0.1100 (15)	
C1	0.25910 (17)	0.8534 (6)	0.1724 (3)	0.0614 (12)	
C2	0.20539 (15)	0.8609 (5)	0.1534 (2)	0.0488 (9)	
C3	0.19023 (15)	0.8011 (7)	0.1997 (2)	0.0596 (11)	
H3	0.2138	0.7596	0.2437	0.071*	
C4	0.14066 (15)	0.8028 (7)	0.1808 (2)	0.0627 (12)	
H4	0.1305	0.7621	0.2119	0.075*	
C5	0.10627 (15)	0.8642 (5)	0.1165 (2)	0.0510 (10)	
C6	0.12064 (16)	0.9276 (6)	0.0699 (2)	0.0563 (10)	
H6	0.0971	0.9721	0.0265	0.068*	
C7	0.17023 (16)	0.9236 (6)	0.0889 (2)	0.0571 (11)	
H7	0.1803	0.9640	0.0577	0.069*	
C8	0.34155 (17)	0.8066 (9)	0.2610 (3)	0.0977 (18)	
H8A	0.3466	0.6959	0.2404	0.146*	
H8B	0.3601	0.7958	0.3107	0.146*	
H8C	0.3528	0.9148	0.2467	0.146*	
N2	−0.04641 (12)	0.3799 (4)	0.12080 (17)	0.0549 (8)	
H2A	−0.0469	0.2835	0.1461	0.082*	
H2B	−0.0329	0.4794	0.1481	0.082*	
H2C	−0.0284	0.3495	0.1008	0.082*	
O3	−0.27704 (11)	0.5652 (5)	−0.06223 (17)	0.0754 (9)	
O4	−0.25318 (15)	0.6482 (7)	−0.1379 (2)	0.1235 (17)	
C9	−0.24390 (17)	0.5845 (7)	−0.0834 (2)	0.0649 (12)	
C10	−0.19295 (13)	0.5235 (6)	−0.03008 (18)	0.0475 (9)	
C11	−0.15324 (15)	0.5639 (6)	−0.0409 (2)	0.0530 (10)	
H11	−0.1591	0.6244	−0.0814	0.064*	
C12	−0.10518 (13)	0.5155 (5)	0.00777 (19)	0.0460 (9)	
H12	−0.0785	0.5435	0.0007	0.055*	
C13	−0.09772 (13)	0.4248 (5)	0.06700 (18)	0.0412 (8)	
C14	−0.13659 (14)	0.3806 (5)	0.07820 (19)	0.0484 (9)	
H14	−0.1306	0.3179	0.1185	0.058*	
C15	−0.18453 (14)	0.4296 (6)	0.0293 (2)	0.0538 (10)	
H15	−0.2112	0.3995	0.0363	0.065*	
C16	−0.32747 (17)	0.6233 (8)	−0.1111 (3)	0.100 (2)	
H16A	−0.3402	0.5467	−0.1521	0.150*	
H16B	−0.3274	0.7526	−0.1234	0.150*	
H16C	−0.3483	0.6091	−0.0903	0.150*	

### Atomic displacement parameters (Å^2^)

**Table:**

	*U*^11^	*U*^22^	*U*^33^	*U*^12^	*U*^13^	*U*^23^
Sn1	0.0315 (2)	0.0292 (2)	0.0350 (2)	0.000	0.01734 (15)	0.000
Cl1	0.0388 (5)	0.0417 (5)	0.0498 (5)	−0.0108 (4)	0.0187 (4)	0.0027 (4)
Cl2	0.0430 (5)	0.0531 (6)	0.0319 (5)	0.0061 (4)	0.0138 (4)	0.0043 (4)
Cl3	0.0441 (5)	0.0385 (5)	0.0611 (6)	0.0090 (4)	0.0322 (5)	0.0006 (4)
Cl4	0.0609 (7)	0.0948 (9)	0.0812 (8)	0.0232 (6)	0.0452 (6)	0.0309 (7)
N1	0.054 (2)	0.094 (3)	0.063 (2)	0.0182 (18)	0.0344 (19)	0.021 (2)
O1	0.0490 (18)	0.115 (3)	0.080 (2)	0.0101 (17)	0.0348 (18)	−0.003 (2)
O2	0.084 (3)	0.174 (4)	0.107 (3)	0.001 (2)	0.074 (3)	0.016 (3)
C1	0.063 (3)	0.055 (3)	0.086 (4)	−0.006 (2)	0.052 (3)	−0.008 (2)
C2	0.056 (2)	0.045 (2)	0.060 (3)	0.0003 (17)	0.040 (2)	−0.0021 (18)
C3	0.051 (2)	0.083 (3)	0.052 (2)	0.013 (2)	0.031 (2)	0.017 (2)
C4	0.056 (3)	0.091 (3)	0.056 (3)	0.019 (2)	0.038 (2)	0.028 (2)
C5	0.052 (2)	0.056 (3)	0.055 (2)	0.0110 (18)	0.034 (2)	0.0093 (19)
C6	0.063 (3)	0.065 (3)	0.047 (2)	0.009 (2)	0.032 (2)	0.0141 (19)
C7	0.072 (3)	0.062 (3)	0.056 (3)	0.003 (2)	0.046 (2)	0.008 (2)
C8	0.053 (3)	0.114 (4)	0.128 (5)	0.012 (3)	0.046 (3)	−0.002 (4)
N2	0.0470 (19)	0.057 (2)	0.052 (2)	0.0027 (15)	0.0173 (16)	−0.0006 (16)
O3	0.0418 (16)	0.094 (2)	0.075 (2)	0.0087 (16)	0.0164 (16)	−0.0012 (18)
O4	0.081 (3)	0.201 (5)	0.063 (2)	0.034 (3)	0.015 (2)	0.051 (3)
C9	0.055 (3)	0.073 (3)	0.047 (3)	0.008 (2)	0.010 (2)	−0.002 (2)
C10	0.046 (2)	0.053 (2)	0.039 (2)	0.0024 (17)	0.0163 (18)	−0.0014 (18)
C11	0.063 (3)	0.055 (2)	0.041 (2)	0.0008 (19)	0.025 (2)	0.0055 (18)
C12	0.046 (2)	0.046 (2)	0.051 (2)	−0.0042 (17)	0.0277 (19)	0.0003 (18)
C13	0.040 (2)	0.042 (2)	0.038 (2)	0.0015 (15)	0.0161 (16)	−0.0023 (16)
C14	0.048 (2)	0.056 (2)	0.040 (2)	0.0017 (18)	0.0207 (18)	0.0097 (17)
C15	0.042 (2)	0.070 (3)	0.048 (2)	−0.0043 (19)	0.0216 (19)	0.004 (2)
C16	0.039 (3)	0.114 (5)	0.103 (4)	0.013 (3)	0.002 (3)	−0.023 (3)

### Geometric parameters (Å, °)

**Table:**

Sn1—Cl1	2.4131 (8)	C8—H8A	0.9600
Sn1—Cl1^i^	2.4131 (8)	C8—H8B	0.9600
Sn1—Cl2^i^	2.4305 (9)	C8—H8C	0.9600
Sn1—Cl2	2.4305 (9)	N2—C13	1.471 (4)
Sn1—Cl3	2.4315 (8)	N2—H2A	0.8900
Sn1—Cl3^i^	2.4315 (8)	N2—H2B	0.8900
N1—C5	1.473 (5)	N2—H2C	0.8900
N1—H1A	0.8900	O3—C9	1.320 (5)
N1—H1B	0.8900	O3—C16	1.448 (5)
N1—H1C	0.8900	O4—C9	1.185 (5)
O1—C1	1.314 (6)	C9—C10	1.489 (5)
O1—C8	1.434 (5)	C10—C15	1.380 (5)
O2—C1	1.197 (5)	C10—C11	1.384 (5)
C1—C2	1.492 (5)	C11—C12	1.377 (5)
C2—C7	1.373 (6)	C11—H11	0.9300
C2—C3	1.384 (5)	C12—C13	1.375 (5)
C3—C4	1.368 (5)	C12—H12	0.9300
C3—H3	0.9300	C13—C14	1.371 (5)
C4—C5	1.362 (5)	C14—C15	1.378 (5)
C4—H4	0.9300	C14—H14	0.9300
C5—C6	1.380 (5)	C15—H15	0.9300
C6—C7	1.369 (5)	C16—H16A	0.9600
C6—H6	0.9300	C16—H16B	0.9600
C7—H7	0.9300	C16—H16C	0.9600
			
Cl1—Sn1—Cl1^i^	89.79 (5)	C2—C7—H7	119.5
Cl1—Sn1—Cl2^i^	89.66 (3)	O1—C8—H8A	109.5
Cl1^i^—Sn1—Cl2^i^	90.63 (3)	O1—C8—H8B	109.5
Cl1—Sn1—Cl2	90.63 (3)	H8A—C8—H8B	109.5
Cl1^i^—Sn1—Cl2	89.66 (3)	O1—C8—H8C	109.5
Cl2^i^—Sn1—Cl2	179.58 (4)	H8A—C8—H8C	109.5
Cl1—Sn1—Cl3	90.88 (3)	H8B—C8—H8C	109.5
Cl1^i^—Sn1—Cl3	179.00 (3)	C13—N2—H2A	109.5
Cl2^i^—Sn1—Cl3	88.64 (3)	C13—N2—H2B	109.5
Cl2—Sn1—Cl3	91.06 (3)	H2A—N2—H2B	109.5
Cl1—Sn1—Cl3^i^	179.00 (3)	C13—N2—H2C	109.5
Cl1^i^—Sn1—Cl3^i^	90.88 (3)	H2A—N2—H2C	109.5
Cl2^i^—Sn1—Cl3^i^	91.06 (3)	H2B—N2—H2C	109.5
Cl2—Sn1—Cl3^i^	88.64 (3)	C9—O3—C16	115.8 (4)
Cl3—Sn1—Cl3^i^	88.45 (4)	O4—C9—O3	124.0 (4)
C5—N1—H1A	109.5	O4—C9—C10	123.4 (5)
C5—N1—H1B	109.5	O3—C9—C10	112.6 (4)
H1A—N1—H1B	109.5	C15—C10—C11	119.7 (3)
C5—N1—H1C	109.5	C15—C10—C9	121.8 (4)
H1A—N1—H1C	109.5	C11—C10—C9	118.5 (4)
H1B—N1—H1C	109.5	C12—C11—C10	120.8 (4)
C1—O1—C8	117.0 (4)	C12—C11—H11	119.6
O2—C1—O1	123.1 (4)	C10—C11—H11	119.6
O2—C1—C2	123.4 (5)	C13—C12—C11	118.3 (3)
O1—C1—C2	113.5 (4)	C13—C12—H12	120.9
C7—C2—C3	119.2 (4)	C11—C12—H12	120.9
C7—C2—C1	120.1 (4)	C14—C13—C12	121.8 (3)
C3—C2—C1	120.6 (4)	C14—C13—N2	119.2 (3)
C4—C3—C2	120.1 (4)	C12—C13—N2	118.9 (3)
C4—C3—H3	120.0	C13—C14—C15	119.5 (4)
C2—C3—H3	120.0	C13—C14—H14	120.3
C5—C4—C3	119.9 (4)	C15—C14—H14	120.3
C5—C4—H4	120.0	C14—C15—C10	119.8 (4)
C3—C4—H4	120.0	C14—C15—H15	120.1
C4—C5—C6	121.0 (4)	C10—C15—H15	120.1
C4—C5—N1	119.9 (3)	O3—C16—H16A	109.5
C6—C5—N1	119.0 (4)	O3—C16—H16B	109.5
C7—C6—C5	118.8 (4)	H16A—C16—H16B	109.5
C7—C6—H6	120.6	O3—C16—H16C	109.5
C5—C6—H6	120.6	H16A—C16—H16C	109.5
C6—C7—C2	121.0 (3)	H16B—C16—H16C	109.5
C6—C7—H7	119.5		

Symmetry codes: (i) −*x*, *y*, −*z*+1/2.

### Hydrogen-bond geometry (Å, °)

**Table:**

*D*—H···*A*	*D*—H	H···*A*	*D*···*A*	*D*—H···*A*
N1—H1A···Cl3	0.89	2.78	3.659 (4)	170
N2—H2B···Cl3	0.89	2.71	3.479 (3)	145
N2—H2C···Cl4	0.89	2.21	3.098 (4)	177
N1—H1B···Cl4^ii^	0.89	2.29	3.155 (4)	165
N1—H1C···Cl4^iii^	0.89	2.22	3.092 (4)	166
N2—H2A···Cl1^iv^	0.89	3.01	3.482 (3)	115
C3—H3···O4^v^	0.93	2.39	3.148 (6)	139
C15—H15···O2^vi^	0.93	2.38	3.130 (5)	138

Symmetry codes: (ii) −*x*, −*y*+1, −*z*; (iii) *x*, *y*+1, *z*; (iv) *x*, *y*−1, *z*; (v) *x*+1/2, −*y*+3/2, *z*+1/2; (vi) *x*−1/2, *y*−1/2, *z*.
